# Zepto-molar electrochemical detection of *Brucella* genome based on gold nanoribbons covered by gold nanoblooms

**DOI:** 10.1038/srep18060

**Published:** 2015-12-14

**Authors:** Amid Rahi, Naghmeh Sattarahmady, Hossein Heli

**Affiliations:** 1 Department of Nanomedicine, School of Advanced Medical Sciences and Technologies, Shiraz University of Medical Sciences, Shiraz, Iran; 2 Nanomedicine and Nanobiology Research Center, Shiraz University of Medical Sciences, Shiraz, Iran; 3 Department of Medical Physics, School of Medicine, Shiraz University of Medical Sciences, Shiraz, Iran

## Abstract

Gold nanoribbons covered by gold nanoblooms were sonoelectrodeposited on a polycrystalline gold surface at −1800 mV (vs. AgCl) with the assistance of ultrasound and co-occurrence of the hydrogen evolution reaction. The nanostructure, as a transducer, was utilized to immobilize a *Brucella*-specific probe and fabrication of a genosensor, and the process of immobilization and hybridization was detected by electrochemical methods, using methylene blue as a redox marker. The proposed method for detection of the complementary sequence, sequences with base-mismatched (one-, two- and three-base mismatches), and the sequence of non-complementary sequence was assayed. The fabricated genosensor was evaluated for the assay of the bacteria in the cultured and human samples without polymerase chain reactions (PCR). The genosensor could detect the complementary sequence with a calibration sensitivity of 0.40 μA dm^3^ mol^−1^, a linear concentration range of 10 zmol dm^−3^ to 10 pmol dm^−3^, and a detection limit of 1.71 zmol dm^−3^.

The quality of life is closely linked to the ability to control the diseases and quality of our environment. In this regard, continuous, fast and sensitive monitoring techniques are required to control the key parameters. In recent years, new technologies have been developed to improve the clinical diagnostics, personal medicine, and environmental control^1^. Molecular diagnostics based on the analysis of special gene sequences have offered highly sensitive methods for detection of pathogenic microorganisms^2^. In this context, the development of novel DNA based biosensors for highly sensitive and selective detection is useful^3^, because these devices have many applications in identification of the genetic risk factors, medical diagnostics, and environmental monitoring^4^. Among different DNA based biosensors, electrochemical genosensors have been greatly developed due to the high sensitivity, low cost, fast response time, and miniaturization and automation capabilities^5^.

The developments in the field of nanotechnology provide promising opportunities to design and fabricate novel biosensors, which have been widely used for DNA detection^6^. Nanomaterials have been widely employed in biosensing applications with advantages such as high surface area, enhanced amplification of readout system, enhanced electronic properties and electrocatalytic activity as well as biologically-matched scale^7^,^8^,^9^. Gold nanostructures owing to the unique physical and chemical properties are useful to fabricate transducers of sensors and biosensors^10^. Gold nanostructures are biocompatible and can be readily tuned by changing their size, morphology and surrounding chemical entity^11^. In addition, gold nanostructures can offer multifunctional surfaces with a wide range of organic or biological ligands for selective binding^6^ and provide an excellent platform to fabricate other metallic nanostructures^12^,^13^,^14^. All these properties make gold nanostructures proper candidates to fabricate novel sensing strategies with improved sensitivity, stability, and selectivity^15^.

Brucellosis is a worldwide bacterial zoonosis and an important cause of human suffering and economic loss^16^. Brucellosis infections are almost invariably transmitted to people by direct or indirect contact with infected animals or their products. In particular, consumption of unpasteurized milk and dairy products is one of the most important sources of brucellosis infections^17^. *Brucella* organisms are also biological warfare agents^18^. The etiological agents of brucellosis are various *Brucella* species.

Nowadays, *Brucella* isolation by bacterial culture is still a gold standard method that confirms the existence of the causative agent^19^. Other techniques such as complement fixation test, serum agglutination test, Rose Bengal plate test and polymerase chain reactions (PCR) are used as supporting methods^20^,^21^. However, these methods have limitations such as requiring complicated sample pretreatment, being time consuming and needing highly qualified personnel. Moreover, most of these methods are only adapted for the qualitative or semi-quantitative detection of *Brucella* species, which do not meet the requirements for the rapid and accurate identification of brucellosis; it delays the introduction of efficient remedial measures. Several immunosensors for detection of *Brucella* were also reported^22^,^23^. However, most of these sensors are label-dependent and require labeling of biomolecules to convert the antibody/antigen interaction into detectable (optical or electrochemical) signals. Therefore, development of rapid, inexpensive, and easy-to-use methodologies for detection of *Brucella* has attracted considerable interest.

Figure 1 shows field emission scanning electron microscopy (FESEM) images of gold nanoribbons covered by gold nanoblooms with different magnifications. The nanostructure has a complex morphology. At low magnification, it comprises clung ribbons which are partly covered by blooms. At higher magnifications, the images show nanoparticles of gold deposited on the connected smooth-surface nanoribbons. The blooms appeared whitish with a high contrast. This is due to the electron accumulation on the bloom surfaces during FESEM (surface charge), indicating the weak electrical attachment of the nanoblooms to the nanoribbons and underlying substrate. Therefore, the surface of modified gold disk electrode with gold nanoribbons covered by gold nanoblooms (Au/nAu) has a 3D structure comprising multiple electrically independent ensembles of gold nanoribbons separated by loosely-connected nanoblooms. This structure was formed with the assistance of ultrasound, while hydrogen evolution reaction was performed with a high rate due to the highly negative potential of electrodeposition. Therefore, the mass transport regime was highly complex, and the gold seeds were deposited on a surface with the evolution of hydrogen gas. This special feature of 3D nanoscale structure at the surface of the transducer provide higher surface concentration of bound species (such as methylene blue, MB) to the immobilized DNA (probe oligonucleotide (p-ssDNA) or double stranded DNA (dsDNA))^24^. It also leads to conformation of the immobilized p-ssDNA to be favorable for hybridization with target oligonucleotide (t-ssDNA) promoting the hybridization efficiency^13^. On the other hand, the real surface area for the Au/nAu electrode was obtained as 0.73 cm^−2^, compared to the geometric surface area of 0.0314 cm^−2^, with a roughness factor of 23.2. Therefore, the surface texture of the Au/nAu electrode is a 3D ensemble of nanostructures providing a large overall surface area needed for electrochemical sensing without engagement in the restriction of isolated nanostructures^12^,^25^.

Open circuit potential (OCP) of the Au/nAu electrode during the self-assembling process of p-ssDNA at the surface initially showed a gradual shift towards negative values and reached a pseudo-state value within 8 h (Supplementary material S1). This indicates a dramatic accumulation of p-ssDNA on the Au/nAu electrode surface. Figure 2 represents the fabrication protocol of the genosensor and detection of t-ssDNA.

Figure 3A shows differential pulse voltammograms (DPVs) of MB (as a common redox marker in genosensors^26^) interacted with p-ssDNA using the genosensor, before and after hybridization with different concentrations of t-ssDNA recorded in 20 mmol dm^−3^ Tris-HCl buffer, pH 7.4 solution (Tris). MB can interact with both single stranded DNA (ssDNA) and dsDNA through different modes including electrostatic attractions and intercalation^27^. Under the ionic strength of the solution (see Methods section), MB binding to the free guanine bases in p-ssDNA is favorable^28^ through hydrophobic forces of guanine-fused aromatic rings of MB. This fact is supported by a positive shift in the formal potential of MB on the genosensor surface (compared to the bare gold (Au) electrode surface, Supplementary material S2)^29^,^30^,^31^. The dependency of the MB reduction peak current on the t-ssDNA concentration is shown in Fig. 3B. The plot is linear with a regression equation of y = −(0.3978±0.01816)× − (4.2377±0.2943) in the range of 1.44 × 10^−12^ to 1.44 × 10^−20^ mol dm^−3^ of t-ssDNA with a limit of detection (LOD, 3δ/m) of 1.71 zmol dm^−3^. The LOD obtained here which corresponds to ∼1030 t-ssDNA is one of the lowest values reported for the genosensors and has never been achieved (Supplementary material S3) without using electrocatalytic readout systems and special redox markers.

The surface texture of gold nanoribbons covered by gold nanoblooms provides a 3D ensemble of high surface area rough scaffolds, and a dense p-ssDNA layer. At the same time, it may increase the deflection angle between p-ssDNA with favorable orientations^12^,^32^. Such a structure provides more accessibility for t-ssDNA^33^ and enhanced hybridization kinetics. Alterations in the inter-p-ssDNA interactions and steric effects, and changes in the diffusion regime of MB in equilibrium binding with p-ssDNA or dsDNA may also be included. While linear diffusion is dominated for planar electrodes, for the Au/nAu electrode radial diffusion may occur^24^.

A comparison was made between hybridization of t-ssDNA, one-base mismatched oligonucleotide (1 m-ssDNA), two-base mismatched oligonucleotide (2 m-ssDNA), three-base mismatched oligonucleotide (3 m-ssDNA) and non-complementary sequence oligonucleotide (nc-ssDNA) with p-ssDNA. DPVs of MB interacting with the genosensor before and after hybridization with these sequences recorded in Tris are shown in Figure 4. The peak currents are in the order of I_p-ssDNA_≫_nc-ssDNA_ > I_3m-ssDNA_ > I_2m-ssDNA_ > I_1_ _m-ssDNA_ > I_t-ssDNA_. These results indicate that nc-ssDNA has the minor affinity to hybridize with p-ssDNA, and the mismatched oligonucleotides are distinguishable. Therefore, the genosensor has excellent selectivity and distinguishing ability for the hybridization detection of mismatched target sequences.

In order to inspect the reproducibility of the fabrication of genosensor, it was placed in the Piranha solution for 30s and then re-fabricated (n = 5). DPVs of the genosensor for the repeating fabrication (Supplementary material S4) showed a relative standard deviation (RSD) of 5.4% in the peak current.

To inspect the regeneration of the genosensor, after its hybridization with 1.0 × 10^−13^ mol dm^−3^ t-ssDNA, it was immersed in hot water of 90 °C for 5 min to de-hybridize the formed dsDNA. Then, the genosensor was re-hybridized with the same t-ssDNA concentration (n = 5). DPVs (Supplementary material S5) showed a RSD of 11.2% for the regeneration of the genosensor.

The stability of the genosensor was investigated by recording the peak currents in DPVs for 1.0 × 10^−13^ mol dm^−3^ t-ssDNA over 39 days, while the genosensor was stored in Tris in the refrigerator at 4 °C and measured twice in a day in the same conditions (Supplementary material S6). No regular change was observed at least for 35 days.

In order to detect the *Brucella* genome, we extracted the genome from *Brucella abortus*. After determination of the DNA concentration in the extracted samples, solutions of different genome concentrations were prepared. These solutions were placed at 90 °C for 5 minutes to de-hybridize. Then the solution was analyzed by the genosensor and a calibration curve was plotted (Supplementary material S7). The results confirm that the genosensor can detect the genome of *Brucella* species in a linear range of 1.07 × 10^−14^ to 1.07 × 10^−2^ ng μL^−1^ with a LOD of 2.53 × 10^−15^ ng μL^−1^ (3δ/m).

To evaluate the selectivity of the genosensor to other bacterial genomes, some non-*Brucella* samples were checked as negative controls. These samples included *Pseudomonas aeruginosa*, *Staphylococcus aureus*, *Klebsiella* and *Acinetobacter*. Genomes of these species were extracted and de-hybridized; the two concentrations of 2.0 and 1.1 × 10^−6^ ng μL^−1^ from these samples were analyzed with the genosensor. DPVs recorded for the non-*Brucella* genomic samples (Supplementary material S8) showed slight decrements in the peak currents for both concentrations, indicating selectivity of the genosensor toward *Brucella* species even in the presence of a high concentration of these bacterial genomes.

In order to apply the genosensor for detection of *Brucella* in brucellosis patients, we analyzed the human serum samples. It seems that amplification of bacteria in the blood samples using PCR is needed. Samples without PCR amplification were also analyzed (vide infra). DNA was extracted from (six patients and one healthy) human serum samples, amplified by PCR, de-hybridized, and then analyzed using the genosensor with a concentration of 1.1 × 10^−6^ ng μL^−1^. DPVs obtained using the genosensor for the healthy and patient samples (Supplementary material S9) revealed that the peak current related to the healthy sample was almost the same as p-ssDNA, while other samples represent drastic decrements. Therefore, the genosensor can detect the pathogen in patients after PCR amplification, and can be used in clinical diagnosis.

As for the very low LOD of the genosensor, the detection ability of the genosensor for the human patients without PCR amplification of the genomic DNA in the blood serum was also evaluated. DNA was extracted from a patient’s serum, de-hybridized, and then directly analyzed using the genosensor. Decrement in the peak current for the sample was > 10 × standard deviation of the peak current for p-ssDNA (n = 10). Therefore, this decrement is significant and confirms the presence of *Brucella* in the sample. The results indicated that this label-free genosensor is capable of detecting *Brucella* in the blood samples from patients and does not require the sample to be processed in any way.

In summary, we used a sonoelectrodeposition method to fabricate a 3D nanostructure comprising electrically independent ensembles of gold nanoribbons separated by weak electrical attachment of gold nanoblooms. The nanostructure was checked for immobilization of a *Brucella*-specific probe and detection of hybridization with a variety of sequences with a high sensitivity. The high sensitivity would be related to more favorable conformation and deflection angle of the probe for an efficient hybridization, higher surface concentration of the probe, and/or enhanced diffusion regime. These lead to better display of the entangled target sequences arising from the nanotechnology.

## Methods

Details of materials and methods were provided in Supplementary material S10. A 24 base thiolated oligonucleotide probe (p-ssDNA) was designed based on the common genomic sequence in all the *Brucella* species. t-ssDNA, 1m-ssDNA, 2 m-ssDNA, 3 m-ssDNA, and nc-ssDNA are as follows:

p-ssDNA sequence: 5′ SH-(CH_3_)_6_ TGC CGA TCA CTT AAG GGC CTT CAT 3′;

t-ssDNA sequence: 5′-ATG AAG GCC CTT AAG TGA TCG GCA-3′

nc-ssDNA sequence: 5′-AGA CCA AAA AGG CCA CCC CCG GGT-3′

1m-ssDNA sequence: 5′- ATA AAG GCC CTT AAG TGA TCG GCA-3′

2m-ssDNA sequence: 5′- ATG AAG TAC CTT AAG TGA TCG GCA-3′

3 m-ssDNA sequence: 5′- ATA AAG TCC CTT AAG TAA TCG GCA-3′

The oligonucleotide stock solutions were prepared with 20 mmol dm^−3^ Tris-HCl buffer, pH 7.4 solution (Tris).

An Ag/AgCl, 3 mol dm^−3^ KCl and a bare (Au) or modified gold disk electrode with gold nanoribbons covered by gold nanoblooms (Au/nAu) were used as the reference and working electrodes, respectively.

For preparation of Au/nAu, the Au electrode was placed in the synthesis solutions comprising 5 mmol dm^−3^ HAuCl_4_ + 0.5 mol dm^−3^ KCl and sonoelectrodeposition was performed at −1800 mV for 300 s, while the synthesis solution and also the Au electrode surface were irradiated by ultrasound wave of 45 W power. For OCP measurements, an Au screen-printed electrode was employed.

Immobilization of p-ssDNA probe was performed by dropping 10.0 μL of 10.0 μmol dm^−3^ p-ssDNA solution on the Au/nAu electrode surface and kept refrigerated at 4 °C for 8 h. Then the electrode was further treated with 1.0 mmol dm^−3^ 6-Mercapto-1-hexanol and denoted as the genosensor.

A healthy blood sample and 7 human serum samples with Brucellosis (6 samples after amplification by PCR and one sample without PCR) were analyzed.

## Additional Information

**How to cite this article**: Rahi, A. *et al*. Zepto-molar electrochemical detection of *Brucella* genome based on gold nanoribbons covered by gold nanoblooms. *Sci. Rep*. **5**, 18060; doi: 10.1038/srep18060 (2015).

## Supplementary Material

Supplementary Information

## Figures and Tables

**Figure 1:**
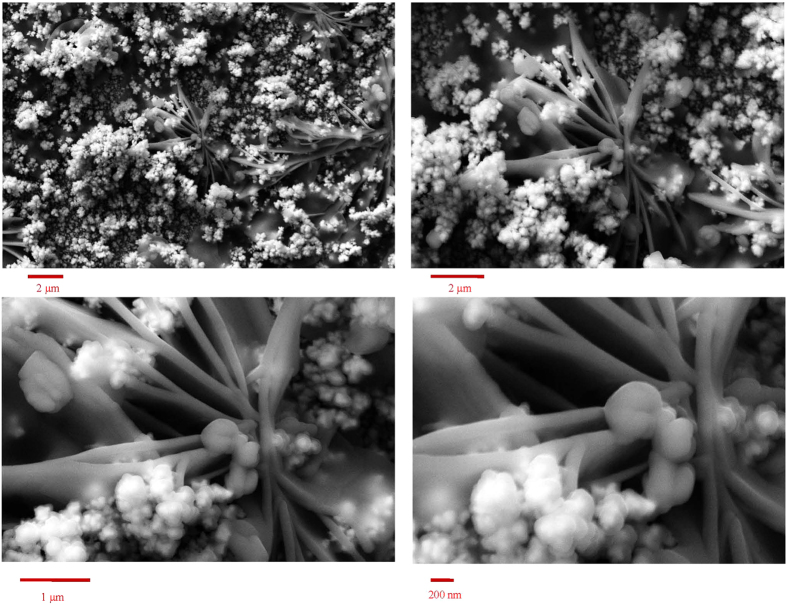
FESEM images of gold nanoribbons covered by gold nanoblooms with different magnifications.

**Figure 2:**
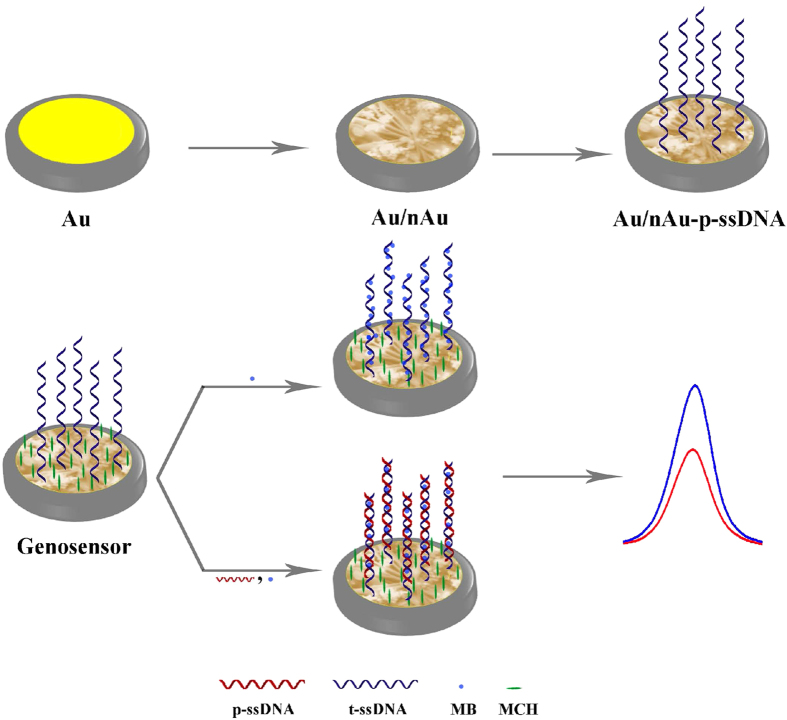
Fabrication protocol of the genosensor and detection of t-ssDNA.

**Figure 3:**
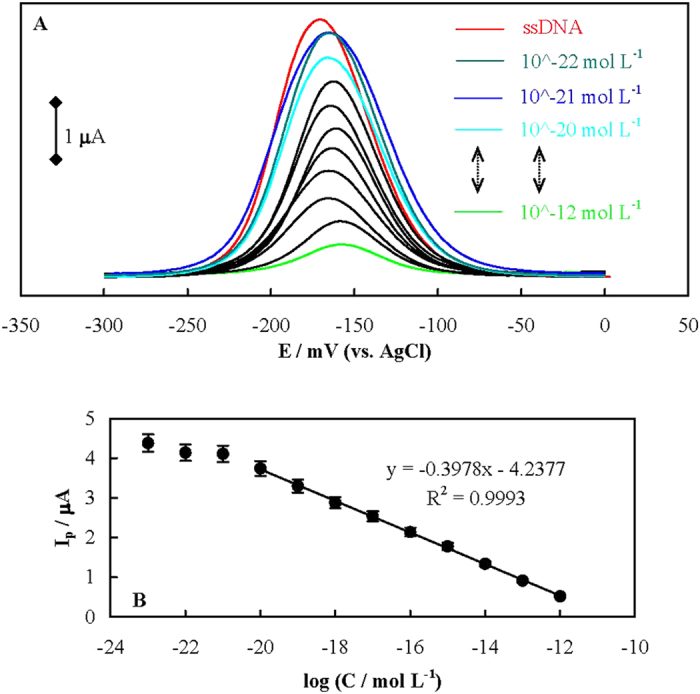
(**A**) DPVs of MB interacted with p-ssDNA using the genosensor, before and after hybridization with different concentrations of t-ssDNA in Tris. (**B**) The dependency of the MB reduction peak current on the t-ssDNA concentration.

**Figure 4:**
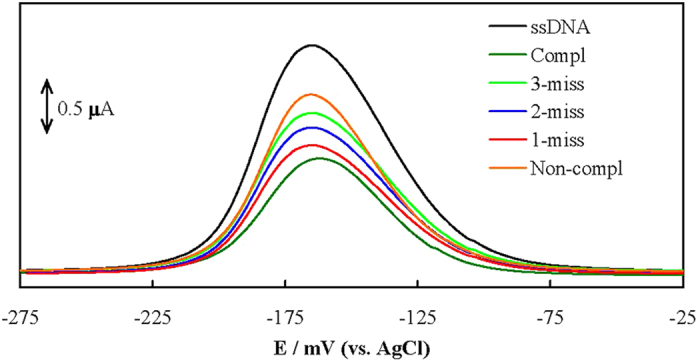
DPVs of MB interacting with the genosensor before and after hybridization with t-ssDNA, 1m-ssDNA, 2 m-ssDNA, 3 m-ssDNA and nc-ssDNA with p-ssDNA.
