# Supplementary material for: Zepto-molar electrochemical detection of Brucella genome based on gold nanoribbons covered by gold nanoblooms
Source: Sci Rep. 2015 Dec 14;5:18060. doi: 10.1038/srep18060 (PMC4677304; doi:10.1038/srep18060)
Supplement: Supplementary Information [file srep18060-s1.pdf]

Supplementary materials

Zepto-molar electrochemical detection of *Brucella* genome based on gold nanoribbons covered by gold nanoblooms

Amid Rahi[1], Naghmeh Sattarahmady[1,2,3]* and Hossein Heli[1,2]*

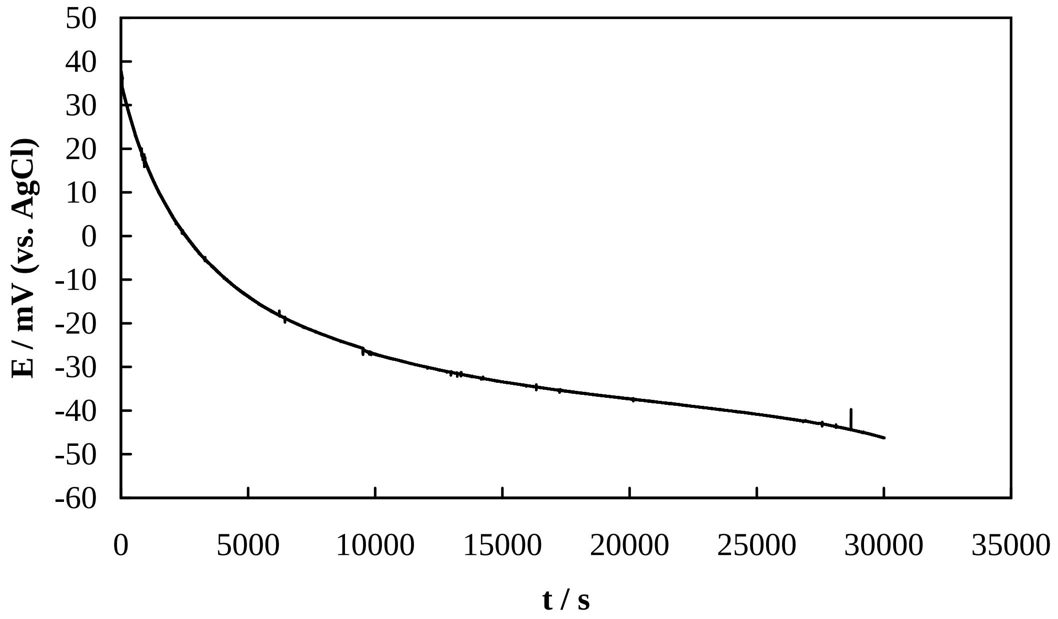

50
40
30
20
10
0
-10
-20
-30
-40
-50
-60
E / mV (vs. AgCl)
0
5000
10000
15000
20000
25000
30000
35000
t / s

S1

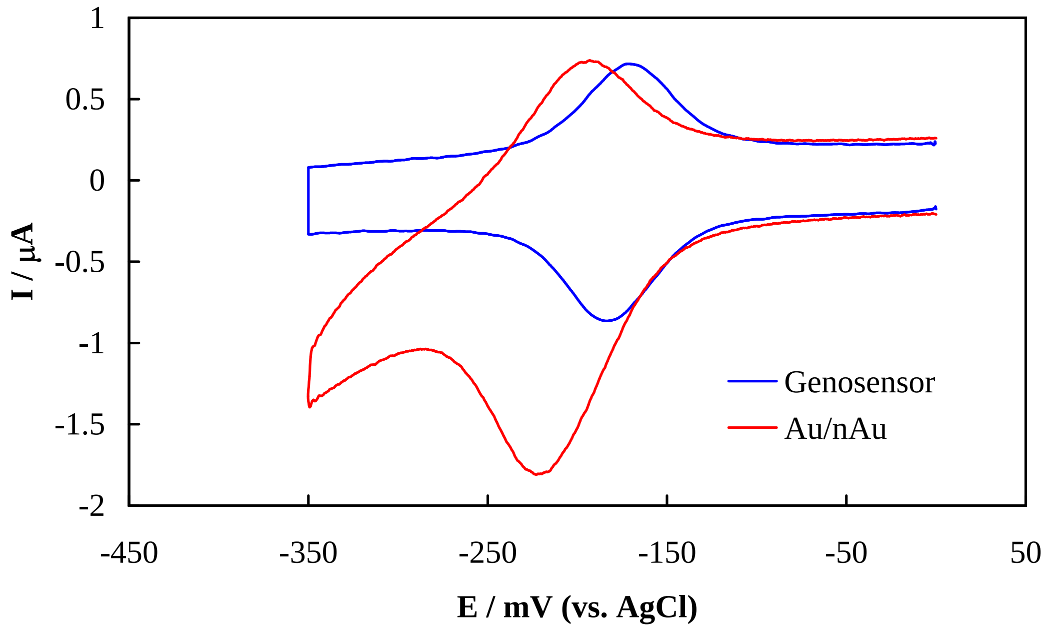

1
0.5
0
-0.5
-1
-1.5
-2
I / μA
-450
-350
-250
-150
-50
50
E / mV (vs. AgCl)
Genosensor
Au/nAu

S2

Supplementary material S3: A comparison of the figure of merit of some genosensors.

Probe sequence	Redox indicator	Substrate	Detection technique	LOD	Linear range	Real Sample	Reference
5'-GTGGTGGGCTGGGCGATCAA-p-(CH2)3-SH	MB	Au	CC, SWV, CV	-	-	*Aeromonas hydrophila*	S1
5'-SH-(CH2)6-ACCACGACGTTGTAAAACGACGGCC AGTCT AT-3'	Ag NPs	GC	ASV	0.5 pM	1-600 pM	-	S2
NH2-C12-5'-GCACCTGACTCCTGTGGAGAAGTCT GCCGT-3'	Label-free	PAN/PAC	EIS	20 nM	50-200 nM	-	S3
5'-NH2-GAGCGCGCAACATTTCAGGTGGA-3'	Ferro/ferricyanide	Au	EIS	-	-	-	S4
5'-NH2-GCCACAAACACCACAAGAGT-3'	Ferro/ferricyanide	Au/PAN	CV, DPV, EIS	0.31 pM	1 pM-1μM	-	S5
5′-SH-(CH2)6-AATGTATAATTGCGCGACTCTAATC-3′	Label-free	Au NPs	DPV	35 fM	0.1 pM-10 nM	-	S6
5'-IATTTTCTTCCTTTTITTC-3'	Label-free	Pencil graphite	CP	0.5 mg/L	10-30 mg/L	BRCA1 breast cancer gene	S7
5-NH2-GAGCGGCGCAACATTTCAGGTCGA-3'	Daunomycin	MWCNTs	DPV	0.1 nM	0.2-50 nM	-	S8
5'-NH2-CAAGACCACCACTTCGAAACC-3'	Label-free	SWCNTs	DPV	0.15 µg/mL	25-40 µg/mL	Calf thymus DNA	S9
5'-NH2-AGTTGCTGATGGTCCTCATGCTGGC-3'	Label-free	GC	EIS	0.50 nM	1-20 nM	-	S10
5'-GCCAGCGTTCAATCTGAGCCATGAT CAAACTCTTCAAATGCCGATTAGGC-(A)6-(CH2)6-SH-3'	Os-C1	Au	Amp, CV	600 fM	1.0-300 pM	-	S11
5'-HS(CH2)6GCGTTCCAAAGGGCAGGA TCATTGA-3'	Os-C2	Au	CV, DPV	6 pmol	5-20 pmol	Helicobacter pylori	S12

5'-GGGGCAGAGCCTCACAACCT-(CH2)3-SH-3'	AQMS	Au	CV, OSWV	0.5 nM	-	-	S13
5′-CGACAGTGGTCCCAAAGA-3′	Ferro/ferricyanide	SWNTs	DPV	20 nM	40-110 nM	-	S14
5'-PO4-GAGCGGCGCAACATTTCAGGTCGA-3'	Daunomycin	Chit/PB/GPE	DPV, CV	0.16 pM	0.21pM-21.2 nM	-	S15
5'-GGGCACTCTTGCCTACGCCATCAGCTCCAACTACCACAAGTTTT-3'	Daunomycin	MWNTs/Z rO_2 NPs/Chit	DPV	75 pM	14.9-93.2 nM	-	S16
5'-HS-TATTAACTTTACTCC-3'	p-Aminophenol	Au	CA, CV	1 pM	-	*E. Coli*	S17
5'-GCCACAAACACCACAAGAGT-3'	MB	Au NPs/PDC	DPV, CV	24 pM	10 nM-10 μM	-	S18
5'-HS-(CH2)6-ACTGCTAGAGATTTTCCACAT-3'	Label-free	B2dMPTS	EIS	5 nM	10 nM-1 μM	-	S19
5′-TGGACGTGGCTTAGCGTATATT-3′	MB	Chit-Co_3O_4-GR	DPV	0.43 pM	1 pM-1 μM	*Staphylococcus aureus*	S20
NH2-C6-5'-AAGCGGAGGATTGACGACTA-3'	Ferro/ferricyanide	PAMAM-Au	EIS	3.8 pM	10-100 pM	-	S21
5'-GAAACACCAATGATATTTTC-3'	Label-free	MWCNTs	EIS	100 pM	20-80 nM	Cystic Fibrosis mutant gene	S22
5'-HS(CH2)6GTATCTACCACAGTAACAAA-3'	Hematoxylin	Au	CV, DPV	3.8 nM	12.5-350.0 nM	Human papilloma virus	S23
5'-SH-(CH2)6-ACCCTTGGGAGGAAGAGACG-3'	hydroquinone	Au NPs	Amp	5.22 nM	0.53-25 nM	-	S24
5'-HS-(CH2)6-GCACGCTAGATGAGTCCGTCCTGCTGCGTGC-3'	Ferro/ferricyanide	Au	DPV, CV	0.167 pM	0.5 pM-50 nM	-	S25
5'-PO4-GGGGGGCCAAGGCCCAGCCCTCACACA-3'	$[Ru(NH_3)_5Cl]PF_6$	NiO_x NPs	EIS, DPV	68 pM	0.4-10 nM	-	S26
5'-(NH2-C6)ATCAATATTTAACAATAATCC-3'	Daunomycin	Au	DPV	0.13 pM	1 pM-0.1 μM	-	S27
5'-TGG AAATCTTTTTTTGAAAGGCTTTGG-3'	MB	poly-Lysine	CV, DPV	4.35 nM	5-100 nM	Bovine papilloma virus	S28

5'-HS-(CH2)6CGATTCGGTACTGG-3'	TMB	Pt NPs/CNTs	CA	0.6 fM	1.0 fM-10 pM	-	S29
5'-ATTGACCGCTGTGTGACGCAACACTCAATTTCTCCAGTGTAGTATTAGGCAATGAAATTGAGTGTTTTTTTTTTTT-(CH2)3-SH-3'	TMB	Au	CV, Amp	13.6 amol	100-100000 pM	-	S30
5'-GCATATGCAAATGGAACACCTCA-3'	MB	Chit-MgO	CV, DPV	35.2 mg/L	0.1-0.5 mg/L	Vibrio cholerae	S31
Cys-O-O-5'-ATGTACCCCATGAGGTCGGC-3'	MB	Au	DPV	9.5 µg/L	10-300 µg/L	Hepatitis C virus	S32
5′-HS-(CH2)6-GTTCTTCTCATCATCGAC-3'	MB	Aloe-like Au	SWV	12 aM	50 aM-1 pM	-	S33
5'-HS-(CH2)6-GGCCATCGTTGAAGATGCCTCTGCC-3'	$[Ru(NH_3)_6]^{3+}$	PAN-Chit-Au NPs	CC	80 aM	0.1-10 fM	-	S34
5'-AATTT-CCCCC-AAATT-3'	PIND-Ru	CPE	CV	1.5 pM	2.5-350 pM	-	S35
5'-GAG-GAG-TTG-GGG-GAG-CAC-ATT-3'	$[Co(phen)_3]^{3+}$	CPE	DPV	15 mg/L	-	Hepatitis B virus	S36
5'-SH(CH2)6-GCACCTGACCATAGAACGGT-3'	MDB	Au	DPV, EIS	20 nM	-	*B. anthracis*	S37
5'-CTT-TTT-CTT-TTT-GTC-CTT-TTT-AGG-CTCTGT-3'-(CH2)3-SH	Formamide	Au-SPE	CV	2.5 pM	2.5-50 pM	SARS Virus	S38
5'-(SH)GCGTTCCAAAGGGCAGGATCATTGA-3'	Adriamycin	Au NPs/rGO	DPV	35 fM	10 nM-0.1 pM	-	S39
5'-NH2-TGGCGGCACATTTGTCACTGCA-3'	MB	Au/GR	DPV, CV	0.29 pM	1 pM-1 µM	*Listeria monocytogenes*	S40
5'-AACCACACAACCTACTACCTCA-3'	MB	ITO	DPV	0.55 pM	1 pM-0.1 µM	-	S41
5'-GAAGCTGGCAACGCTACCGGT-3'	MB	Chit/Fe_3O_4-GR	DPV, EIS	0.36 pM	1 pM-1 µM	Soybean Lectin	S42
5'-NH2(CH2)6TCGATACTCTCCCCGCCCCTTTTGTATCGACG-3'	Ferro/ferricyanide	Alumina nanochannels	DPV, EIS	0.3 pM	1 pM-1 µM	Legionella spp.	S43
5'-CTTTTGTTC-3'	Au-NPs	GR	CV, CA	1 fM	0.1 fM-0.1 nM	BRCA1 gene	S44

5'-SH-(CH2)6-AAAACCCTCCTCAACCCCCT-3'	Adriamycin	Au NRs	DPV	0.35 aM	0.1 fM-1 nM		S45
5'-SH-(CH2)6 TTTTATGTGGCGGATGAGCGGCA-3'	Ferro/ferricyanide	Au	DPV, EIS	8.7 fM	10 fM-1 nM	Enterobacteriaceae	S46
5′-HS-(CH2)6-GCAGGTATGCACAGTGAGTCTGGGCCGTGTCTCAGT-3′		Au	DPV	80 aM	0.1-20 fM	*Bacillus subtilis*	S47
5' -SH-$(CH_3)_{10}$ TGC CGA TCA CTT AAG GGC CTT CAT-3'	MB	Au nanostructure	DPV	1.71 zM	10 zM-1 pM	Human subjects, cultured samples	This work

Abbreviations:

Limit of detection, LOD
Methylene blue, MB
Chronocoulometry, CC
Square wave voltammetry, SWV
Cyclic voltammetry, CV
Anodic stripping voltammetry, ASV
Electrochemical impedance spectroscopy, EIS
Nanoparticles, NP
Polyaniline, PAN
Polyacrylate, PAC
Differential pulse voltammetry (voltammogram), DPV
Chronopotentiometry, CP
Multi wall carbon nanotube, MWNT
Single wall carbon nanotube, SWCNT
Glassy carbon, GC
Amperometry, Amp
N,N'-bis[(3-propyl)imidazole]-1,4,5,8-naphthalene diimide, PIND
PIND-Os, Os-C1

Os(phen)$_2$(phen-dione)]$^{3+/2+}$, Os-C2
Anthraquinonemonosulfonic acid, AQMS
Osteryoung square wave voltammetry, OSWV
Chitosan, Chit
Chronoamperometry, CA
Carbon nanotube, CNT
Carbon paste electrode, CPE
Reduced grapheme oxide, rGO
Graphene, GR
Indium tin oxide, ITO
Nanorods, NRs
Graphene paste electrode, GPE
Prussian blue, PB
Poly-2,6-pyridinedicarboxylic acid, PDC
Bilayer two-dimensional 3-mercaptopropyltrimethoxysilane, B2dMPTS
3,3',5,5' tetramethylbenzidine, TMB
Meldola's blue, MDB
Screen-printed electrode, SPE

References:

s1- Tichoniuk M, Gwiazdowska D, Ligaj M, Filipiak M. Electrochemical detection of foodborne pathogen Aeromonas hydrophila by DNA hybridization biosensor. Biosens Bioelectron 2010;26(4):1618-1623.

s2- Cai H, Xu Y, Zhu N, He P, Fang Y. An electrochemical DNA hybridization detection assay based on a silver nanoparticle label. Analyst 2002;127(6):803-808.

s3- Huiru G, Xiao S, Kian L. Electrochemical impedance sensing of DNA hybridization on conducting polymer film-modified diamond. J Phys Chem B 2005;109(28):13611-13618.

s4- Xu Y, Cai H, He PG, Fang YZ. Probing DNA hybridization by impedance measurement based on CdS□oligonucleotide nanoconjugates. Electroanalysis 2004;16(1□2):150-155.

s5- Feng Y, Yang T, Zhang W, Jiang C, Jiao K. Enhanced sensitivity for deoxyribonucleic acid electrochemical impedance sensor: gold nanoparticle/polyaniline nanotube membranes. Analytica chimica acta 2008;616(2):144-151.

s6- Zhang Y, Huang L. Label-free electrochemical DNA biosensor based on a glassy carbon electrode modified with gold nanoparticles, polythionine, and graphene. Microchimica Acta 2012;176(3-4):463-470.
s7- Wang J, Kawde AN. Pencil-based renewable biosensor for label-free electrochemical detection of DNA hybridization. Analytica chimica acta 2001;431(2):219-224.
s8- Cai H, Cao X, Jiang Y, He P, Fang Y. Carbon nanotube-enhanced electrochemical DNA biosensor for DNA hybridization detection. Anal bioanal chem 2003;375(2):287-293.
s9- Kerman K, Morita Y, Takamura Y, Tamiya E. Escherichia coli single-strand binding protein-DNA interactions on carbon nanotube-modified electrodes from a label-free electrochemical hybridization sensor. Anal Bioanal Chem 2005;381(6):1114-21.
s10- Xu X, Weng X, Liu A, Lin Q, Wang C, Chen W, Lin X. Electrochemical genosensor for detection of human mammaglobin in polymerase chain reaction amplification products of breast cancer patients. Anal Bioanal Chem 2013;405(10):3097-103.
s11- Tansil NC, Xie H, Xie F, Gao Z. Direct detection of DNA with an electrocatalytic threading intercalator. Anal chem 2005;77(1):126-134.
s12- Del Pozo MV, Alonso C, Pariente F, Lorenzo E. DNA biosensor for detection of Helicobacter pylori using phen-dione as the electrochemically active ligand in osmium complexes. Anal chem 2005;77(8):2550-2557.
s13- Wong EL, Gooding JJ. Charge transfer through DNA: a selective electrochemical DNA biosensor. Anal chem 2006;78(7):2138-2144.
s14- Zhang X, Jiao K, Liu S, Hu Y. Readily reusable electrochemical DNA hybridization biosensor based on the interaction of DNA with single-walled carbon nanotubes. Anal chem 2009;81(15):6006-6012.
s15- Bo Y, Wang W, Qi J, Huang S. A DNA biosensor based on graphene paste electrode modified with Prussian blue and chitosan. Analyst 2011;136(9):1946-1951.
s16- Yang Y, Wang Z, Yang M, Li J, Zheng F, Shen G, Yu R. Electrical detection of deoxyribonucleic acid hybridization based on carbon-nanotubes/nano zirconium dioxide/chitosan-modified electrodes. Anal chim acta 2007;584(2):268-274.
s17- Walter A, Wu J, Flechsig GU, Haake DA, Wang J. Redox cycling amplified electrochemical detection of DNA hybridization: Application to pathogen E. coli bacterial RNA. Anal chim acta 2011;689(1):29-33.

s18- Jie Y, Tao Y, Yuanyuan F, Kui J. A DNA electrochemical sensor based on nanogold-modiWed poly-2, 6-pyridinedicarboxylic acid Wlm and detection of PAT gene fragment. Anal Biochem 2007;365(2):24-30.
s19- Fu Y, Yuan R, Xu L, Chai Y, Zhong X, Tang D. Indicator free DNA hybridization detection via EIS based on self-assembled gold nanoparticles and bilayer two-dimensional 3-mercaptopropyltrimethoxysilane onto a gold substrate. Biochem engin j 2005;23(1):37-44.

s20- Xiaowei Q, Hongwei G, Yuanyuan Z, Xiuzhen W, Ying C, Wei S. Electrochemical DNA biosensor with chitosan-Co3O4 nanorod-graphene composite for the sensitive detection of staphylococcus aureus nuc gene sequence. Bioelectrochemistry 2012;88(2):42–47.

s21- Li A, Yang F, Ma Y, Yang X. Electrochemical impedance detection of DNA hybridization based on dendrimer modified electrode. Biosens Bioelectron2007;22(8):1716-1722.

s22- Bonanni A, Esplandiu MJ, del Valle M. Impedimetric genosensing of DNA polymorphism correlated to cystic fibrosis: a comparison among different protocols and electrode surfaces. Biosens Bioelectron 2010;26(4):1245-1251.

s23- Nasirizadeh N, Zare HR, Pournaghi-Azar MH, Hejazi MS. Introduction of hematoxylin as an electroactive label for DNA biosensors and its employment in detection of target DNA sequence and single-base mismatch in human papilloma virus corresponding to oligonucleotide. Biosens Bioelectron 2011;26(5):2638-2644.

s24- Soreta TR, Henry OY, ÓSullivan CK. Electrode surface nanostructuring via nanoparticle electronucleation for signal enhancement in electrochemical genosensors. Biosens Bioelectron 2011;26(9):3962-3966.

s25- Liu Z, Zhang W, Zhu S, Zhang L, Hu L, Parveen S, Xu G. Ultrasensitive signal-on DNA biosensor based on nicking endonuclease assisted electrochemistry signal amplification. Biosens Bioelectron 2011;29(1):215-218.

s26- Noorbakhsh A, Salimi A. Development of DNA electrochemical biosensor based on immobilization of ssDNA on the surface of nickel oxide nanoparticles modified glassy carbon electrode. Biosens Bioelectron 2011;30(1):188-196.

s27- Zhu L, Luo L, Wang Z. DNA electrochemical biosensor based on thionine-graphene nanocomposite. Biosens Bioelectron 2012;35(1):507-511.

s28- Nascimento GA, Souza EV, Campos-Ferreira DS, Arruda MS, Castelletti CH, Wanderley MS, Lima-Filho JL. Electrochemical DNA biosensor for bovine papillomavirus detection using polymeric film on screen-printed electrode. Biosens Bioelectron 2012;38(1):61-66.

s29- Dong XY, Mi XN, Zhang L, Liang TM, Xu JJ, Chen HY. DNAzyme-functionalized Pt nanoparticles/carbon nanotubes for amplified sandwich electrochemical DNA analysis. Biosens Bioelectron 2012;38(1):337-341.

s30- Cai Z, Song Y, Wu Y, Zhu Z, Yang CJ, Chen X. An electrochemical sensor based on label-free functional allosteric molecular beacons for detection target DNA/miRNA. Biosens Bioelectron 2013;15(41):783-8.

s31-Manoj Kumar Md, Azahar Ali Md, Zafaryab VVA, Moshahid M, Ansari ZA, Ansari SG, Bansi D. Biocompatible nanostructured magnesium oxide-chitosan platform for genosensing application. Biosens Bioelectron 2013;45(15):181-188.

s32- Ahour F, Pournaghi-Azar MH, Alipour E, Hejazi MS. Detection and discrimination of recombinant plasmid encoding hepatitis C virus core/E1 gene based on PNA and double-stranded DNA hybridization. Biosens Bioelectron 2013;15(45):287-91.

s33- Shi L, Chu Z, Liu Y, Jin W, Chen X. Facile synthesis of hierarchically aloe-like gold micro/nanostructures for ultrasensitive DNA recognition. Biosens Bioelectron 2013;49(3):184-191.

s34- Ren R, Leng C, Zhang S. A chronocoulometric DNA sensor based on screen-printed electrode doped with ionic liquid and polyaniline nanotubes. Biosens Bioelectronics 2010;25(9):2089-2094.
s35- Tansil NC, Xie F, Xie H, Gao Z. An ultrasensitive nucleic acid biosensor based on the catalytic oxidation of guanine by a novel redox threading intercalator. Chem Commun 2005;12(8):1064-1066.
s36- Erdem A, Kerman K, Meric B, Akarca US, Ozsoz M. DNA electrochemical biosensor for the detection of short DNA sequences related to the hepatitis B virus. Electroanalysis 1999;11(8):586-587.
s37- Kara P, Meric B, Ozsoz M. Application of impedimetric and voltammetric genosensor for detection of a biological warfare: Anthrax. Electroanalysis 2008;20(24):2629-2634.
s38- Mart?nez□Paredes G, Gonz?lez□Garc?a MB, Costa□Garc?a A. Genosensor for SARS Virus Detection Based on Gold Nanostructured Screen□Printed Carbon Electrodes. Electroanalysis 2009;21(3-5):379-385.
s39- Zhang Y, Jiang W. Decorating graphene sheets with gold nanoparticles for the detection of sequence-specific DNA. Electrochimica Acta 2012;71(3):239-245.
s40- Sun W, Qi X, Zhang Y, Yang H, Gao H, Chen Y, Sun Z. Electrochemical DNA biosensor for the detection of Listeria monocytogenes with dendritic nanogold and electrochemical reduced graphene modified carbon ionic liquid electrode. Electrochimica Acta 2012;85:145-151.
s41- Du D, Guo S, Tang L, Ning Y, Yao Q, Zhang GJ. Graphene-modified electrode for DNA detection via PNA–DNA hybridization. Sens Actuat B Chem 2013;186(5):563-570.
s42- Wei S, Xiaowei Q, Ying C, Shengyun L, Hongwei G. Application of chitosan/Fe3O4 microsphere–graphene composite modified carbon ionic liquid electrode for the electrochemical detection of the PCR product of soybean Lectin gene sequence.Talanta 2011;87(9):106-112.
s43- Rai V, Deng J, Toh CS. Electrochemical nanoporous alumina membrane-based label-free DNA biosensor for the detection of Legionella sp. Talanta 2012;98(13):112-117.
s44- Rasheed PA, Sandhyarani N. Graphene-DNA electrochemical sensor for the sensitive detection of BRCA1 gene. Sens Actuat B Chem 2014;204(12):777-782.
s45- Shi A, Wang J, Han X, Fang X, Zhang Y. A sensitive electrochemical DNA biosensor based on gold nanomaterial and graphene amplified signal. Sens Actuat B Chem 2014;200(10):206-212.
s46- Caihui L, Hua T, Wei C, Li Y, Decai Z, Huangxian J, Shijia D. A sensitiveelectrochemicalDNAbiosensorforspecific detectionof Enterobacteriaceae bacteriabyExonucleaseIII-assisted signal amplification. Biosens Bioelectron 2013;48(4):132-137.
s47- Hu Y, Xu X, Liu Q, Wang L, Lin Z, Chen G. Ultrasensitive Electrochemical Biosensor for Detection of DNA from Bacillus subtilis by Coupling Target-Induced Strand Displacement and Nicking Endonuclease Signal Amplification. Anal chem 2014;86(17): 8785-8790.

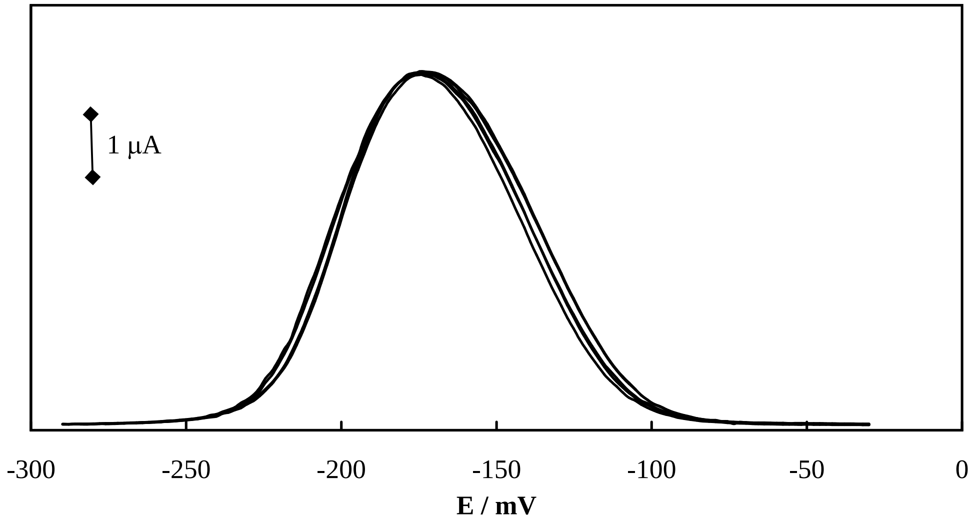

S4

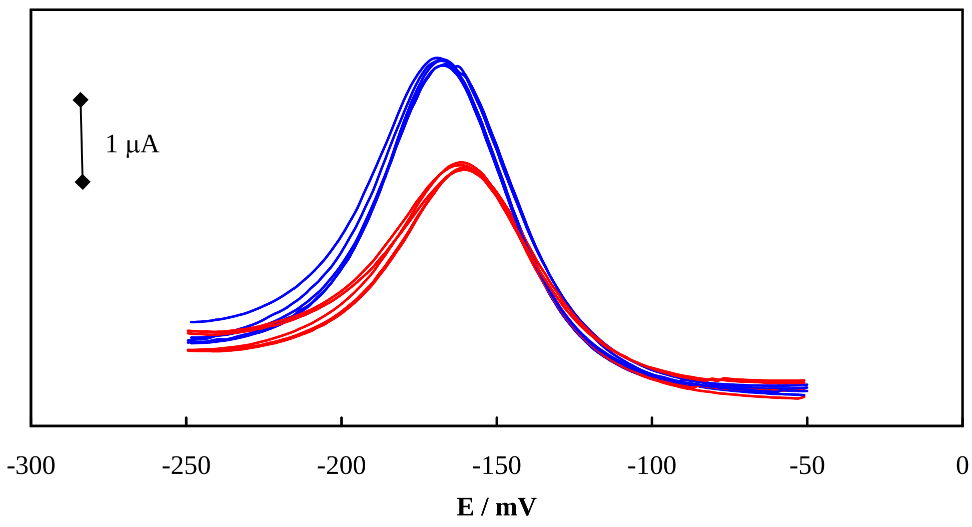

S5

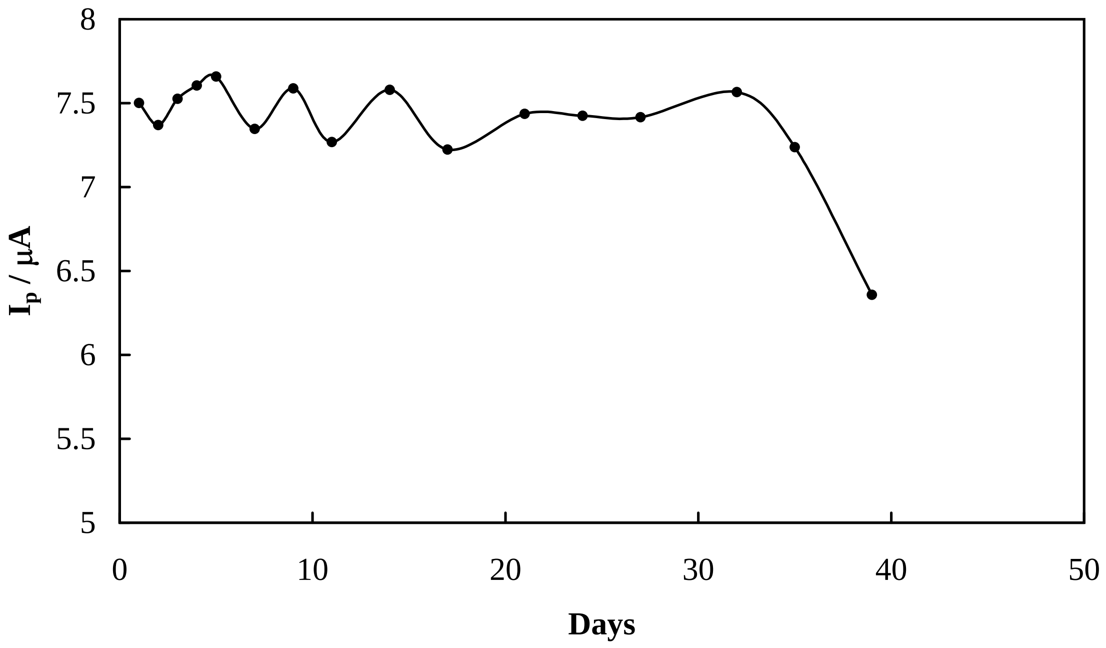

S6

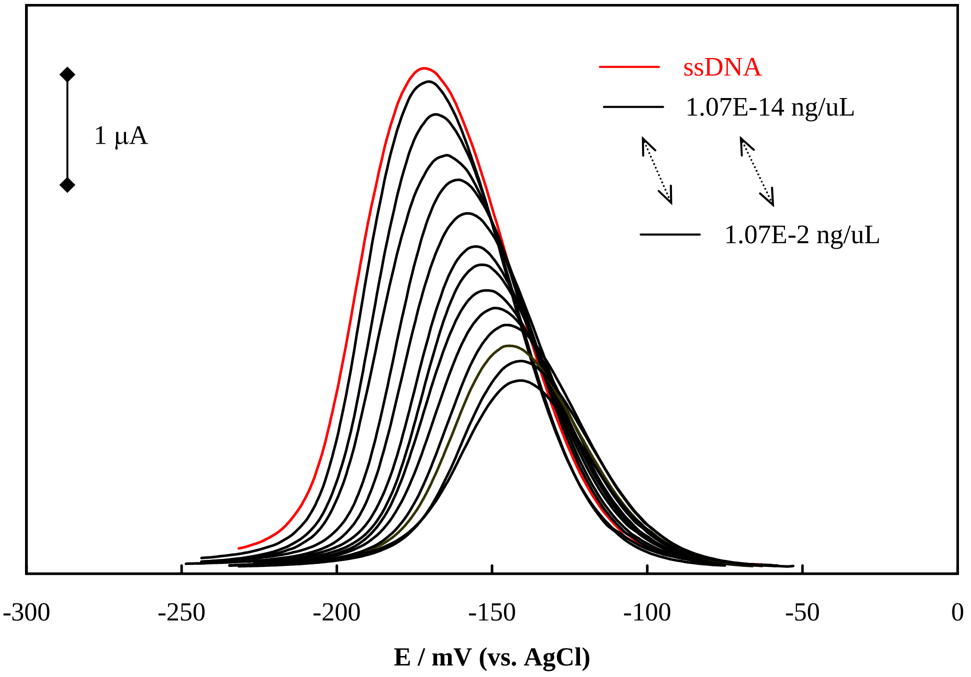

1 μA
ssDNA
1.07E-14 ng/uL
1.07E-2 ng/uL
-300
-250
-200
-150
-100
-50
0
E / mV (vs. AgCl)

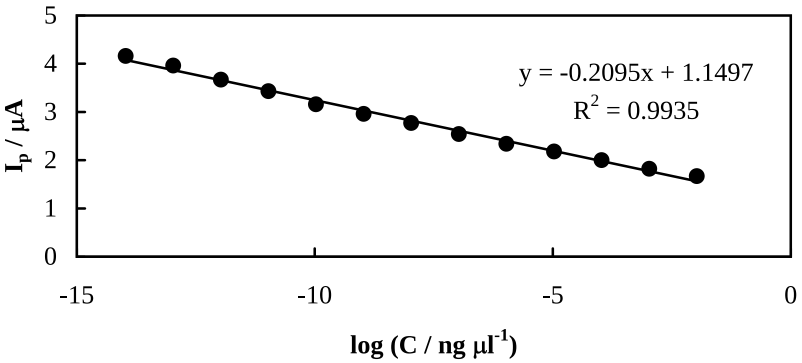

5
4
3
2
1
0
-15
-10
-5
0
I_p / μA
y = -0.2095x + 1.1497
R^2 = 0.9935
log (C / ng μl^{-1})

S7

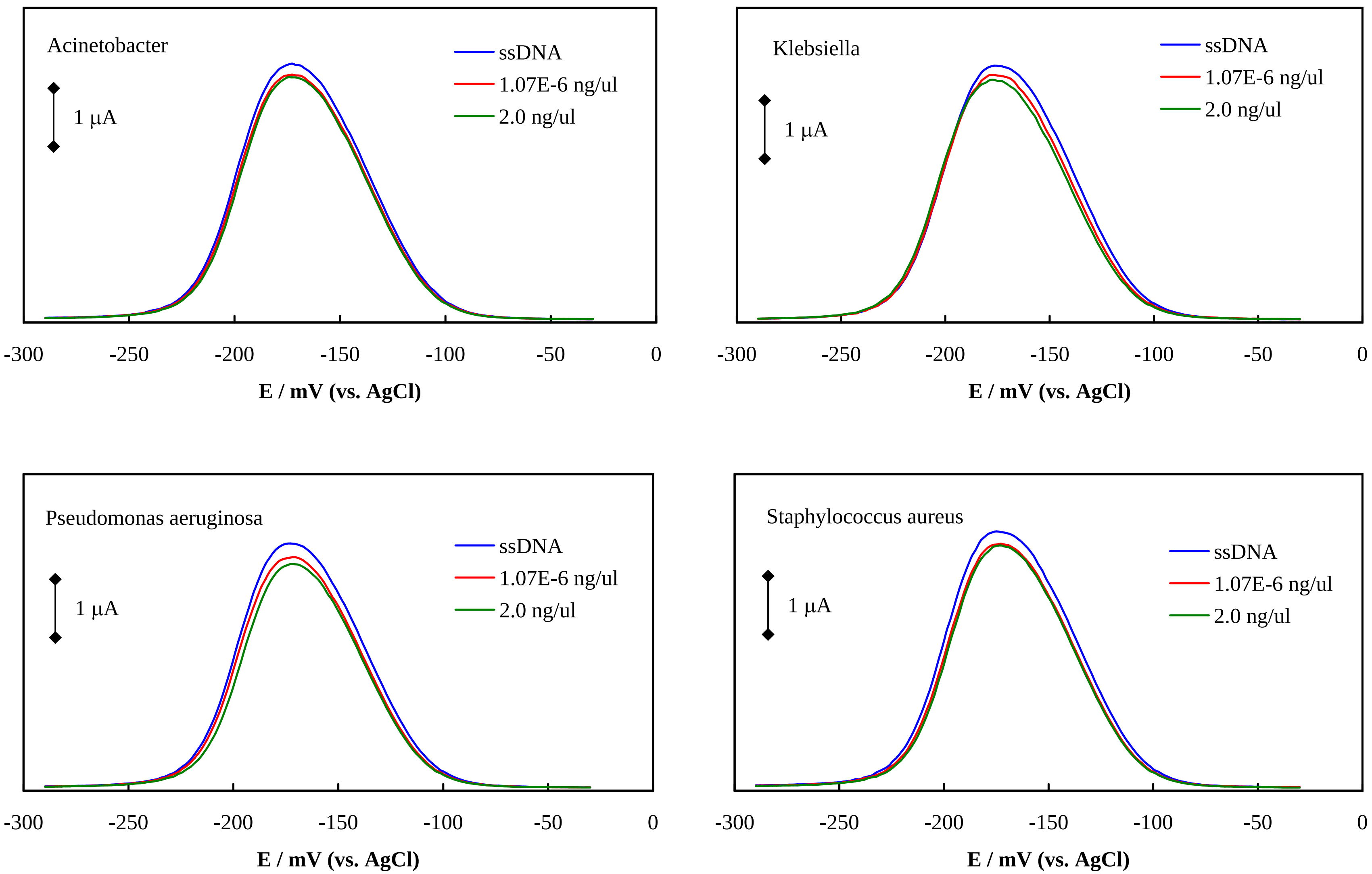
Acinetobacter
ssDNA
1.07E-6 ng/ul
2.0 ng/ul
1 μA
-300 -250 -200 -150 -100 -50 0
E / mV (vs. AgCl)
Klebsiella
ssDNA
1.07E-6 ng/ul
2.0 ng/ul
1 μA
-300 -250 -200 -150 -100 -50 0
E / mV (vs. AgCl)
Pseudomonas aeruginosa
ssDNA
1.07E-6 ng/ul
2.0 ng/ul
1 μA
-300 -250 -200 -150 -100 -50 0
E / mV (vs. AgCl)
Staphylococcus aureus
ssDNA
1.07E-6 ng/ul
2.0 ng/ul
1 μA
-300 -250 -200 -150 -100 -50 0
E / mV (vs. AgCl)

S8

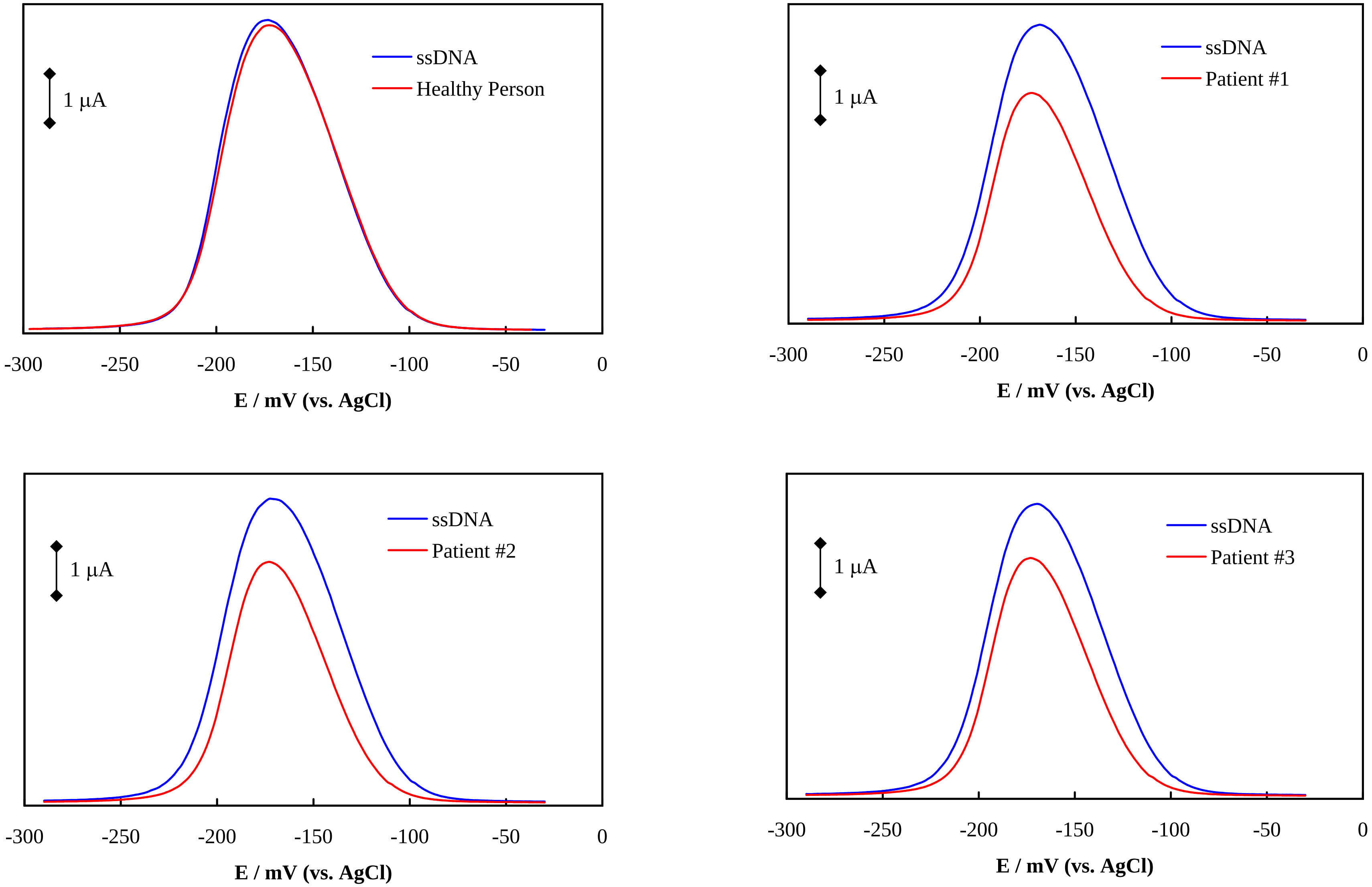
1 μA
ssDNA
Healthy Person
Patient #1
Patient #2
Patient #3
-300
-250
-200
-150
-100
-50
0
E / mV (vs. AgCl)

S9-1

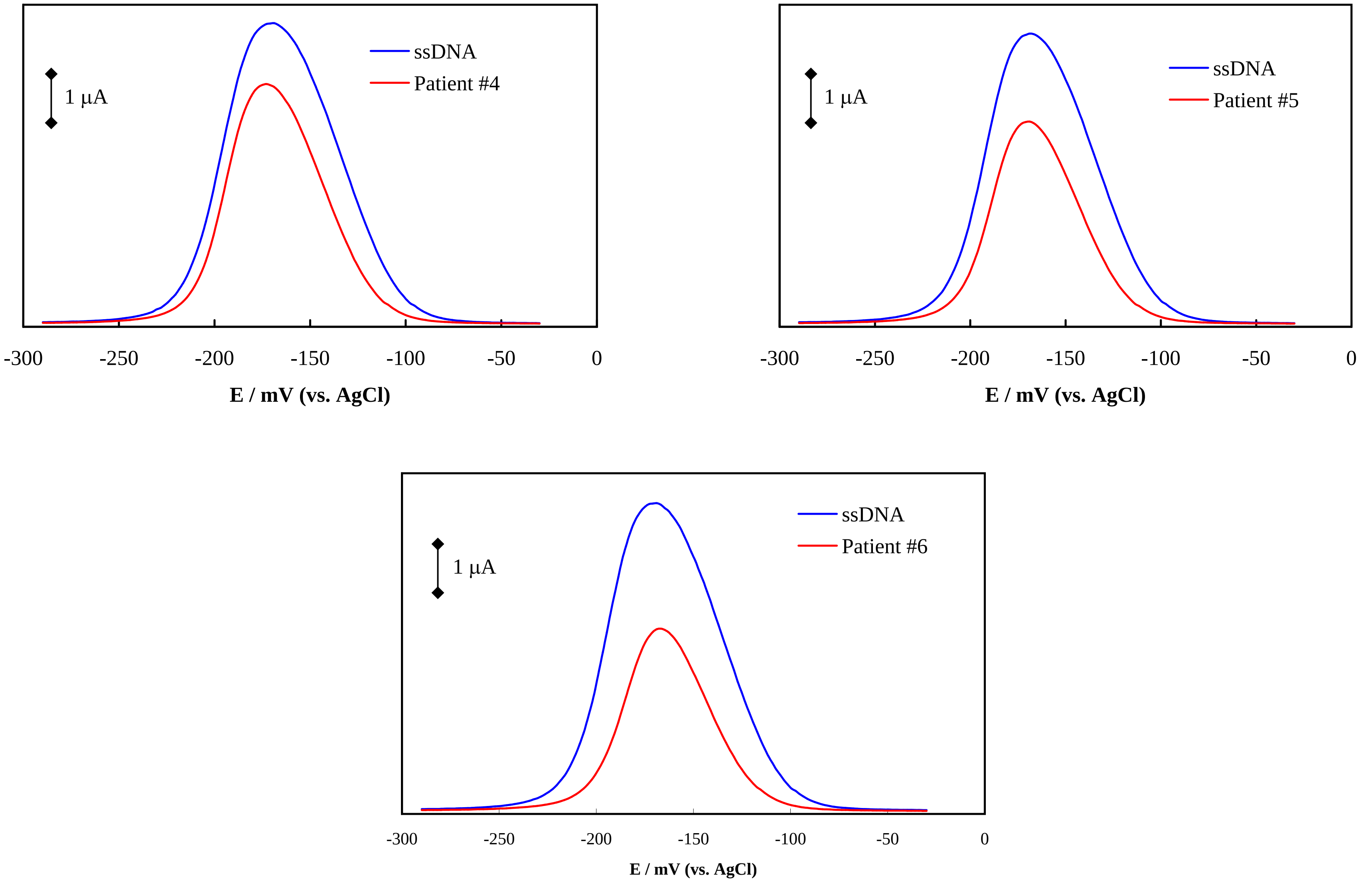
1 μA
ssDNA
Patient #4
-300
-250
-200
-150
-100
-50
0
E / mV (vs. AgCl)
1 μA
ssDNA
Patient #5
-300
-250
-200
-150
-100
-50
0
E / mV (vs. AgCl)
1 μA
ssDNA
Patient #6
-300
-250
-200
-150
-100
-50
0
E / mV (vs. AgCl)

S9-2

Legends of supplementary materials:

S1: Variation of OCP of the Au/nAu electrode during the self-assembling process of p-ssDNA on its surface.

S2: Cyclic voltammograms MB obtained using the genosensor (intercalated MB) and the Au/nAu electrode (… M MB present in the solution phase). The potential sweep rate was 50 mV s^{-1}.

S3: A comparison of the figure of merit of some genosensors.

S4: DPVs of the genosensor recorded for the repeating fabrication.

S5: DPVs recorded for the regeneration of the genosensor. Blue curves show DPVs for the genosensor before hybridization, and red curves show DPVs for the genosensor before re-hybridization.

S6: Variation of the peak current in DPVs for 1.0×10^{-10} mol dm^{-3} t-ssDNA over 39 days to check the stability of the genosensor.

S7: DPVs for MB recorded using the genosensor at different concentrations of the *Brucella* DNA genome (A), and the dependency of the peak current on concentration (B).

S8: DPVs for MB recorded using the genosensor at two concentrations of bacterial genomes of *Pseudomonas aeruginosa*, *Staphylococcus aureus*, *Klebsiella* and *Acinetobacter* (negative controls).

S9: DPVs for MB recorded using the genosensor for the healthy and patient samples.

Details of materials and methods

Materials

All chemicals were of analytical grade from Merck (Germany) or Sigma (USA) and were used without further purification. All solutions were prepared by redistilled water. A 24 base thiolated oligonucleotide probe (probe oligonucleotide, p-ssDNA) was designed based on the common genomic sequence in all the *Brucella* species. p-ssDNA was ordered from Vivantis (Malaysia). A complementary-sequence oligonucleotide (target oligonucleotide, t-ssDNA), one-base mismatched oligonucleotide (1m-ssDNA), two-base mismatched oligonucleotide (2m-ssDNA), three-base mismatched oligonucleotide (3m-ssDNA), and noncomplementary sequence oligonucleotide (nc-ssDNA) were purchased from SinaClon BioScience Co. (Iran). The oligonucleotide sequences are as follows:

p-ssDNA sequence: 5' SH-$(CH_3)_6$ TGC CGA TCA CTT AAG GGC CTT CAT 3';

t-ssDNA sequence: 5'-ATG AAG GCC CTT AAG TGA TCG GCA-3'

nc-ssDNA sequence: 5'-AGA CCA AAA AGG CCA CCC CCG GGT-3'

1m-ssDNA sequence: 5'- ATA AAG GCC CTT AAG TGA TCG GCA-3'

2m-ssDNA sequence: 5'- ATG AAG TAC CTT AAG TGA TCG GCA-3'

3m-ssDNA sequence: 5'- ATA AAG TCC CTT AAG TAA TCG GCA-3'

The oligonucleotide stock solutions were prepared with 20 mmol dm^{-3} Tris-HCl buffer, pH 7.4 solution (Tris) and were kept frozen.

Apparatus

Electrochemical measurements were carried out in a conventional three-electrode cell powered by a μ-Autolab potentiostat/galvanostat (the Netherlands). An Ag/AgCl, 3

mol dm^{-3} KCl, a platinum wire, and a bare (Au, 2 mm of diameter) or modified gold disk electrode with gold nanoribbons covered by gold nanoblooms (Au/nAu) were used as the reference, counter and working electrodes, respectively. The system was run on a PC by GPES 4.9 software.

DNA samples for PCR amplification were extracted from *Brucella* strains, *Pseudomonas aeruginosa*, *Staphylococcus aureus*, *Klebsiella* and *Acinetobacter*. The PCR reaction was performed on an Eppendorf Mastercycler Gradient PCR system (USA). To measure the concentration of the extracted DNA samples, Thermo Scientific NanoDrop 2000c (USA) was employed.

In order to obtain information about the morphology and size of gold nanostructures, field emission scanning electron microscopy (FESEM) was performed using a Zeiss, Sigma-IGMA/VP (Germany). The samples were coated by a 2-5-nm thin film of gold through sputtering.

Preparation of the modified electrode

Before sonoelectrodeposition of gold nanoribbons was covered by gold nanoblooms, the Au electrode was polished on a sand papers and then on a polishing pad with 50 nm-alumina powder lubricated by glycerin. Polishing was continued to attain a mirror-like Au electrode surface. The electrode was then cleaned by immersion in a 1:3 water/ethanol mixture and ultrasonication for 8 min in an ultrasound bath. The electrode was further electropolished by immersion in a 500 mmol dm^{-3} H_2SO_4 solution and applying potential in the range of cathodic to anodic edges of the electrolyte stability in a regime of cyclic voltammetry for 20 consecutive cycles. Upon this pretreatment, clean

and stable Au electrode surface was attained. The Au electrode was then placed in the cell containing the synthesis solutions comprising 5 mmol dm^{-3} $HAuCl_4$ + 0.5 mol dm^{-3} KCl. Sonoelectrodeposition of gold nanoribbons covered by gold nanoblooms was performed at -1800 mV for 300 s, while the synthesis solution and also the Au electrode surface were irradiated by ultrasound wave of 45 W power. The modified electrode was then rinsed thoroughly with distilled water. For preparation of the Au/nAu electrode for open circuit potential (OCP) measurements, an Au screen-printed electrode was directly transferred to the synthesis solutions and the sonoelectrodedeposition process was done similarly.

Immobilization of p-ssDNA

Immobilization of p-ssDNA probe was performed by dropping 10.0 μL of 10.0 μmol dm^{-3} p-ssDNA solution dissolved in Tris on the Au/nAu electrode surface and kept refrigerated at 4 °C for 8 h. Then the electrode was rinsed with Tris. The DNA-modified Au/nAu electrode was further treated with 1.0 mmol dm^{-3} 6-Mercapto-1-hexanol at room temperature for 30 min to obtain a well aligned p-ssDNA monolayer. Then the electrode was washed again with Tris and double distilled water, respectively, to remove unspecific absorbed p-ssDNA. The obtained electrode was denoted as the genosensor.

Hybridization

The hybridization process was performed by immersing the genosensor into Tris containing various concentrations of t-ssDNA for 1 h at 37 °C. Then, the electrode was rinsed with Tris to remove the un-hybridized t-ssDNA, and then it was incubated in a

solution containing Tris + NaCl (20 mmol dm^{-3}) + methylene blue (MB, 20 μmol dm^{-3}) for 5 min at 37 °C. Finally, the electrode was rinsed with Tris to remove the physically absorbed MB.

Electrochemical measurements

The real surface area of the Au/nAu electrode was measured electrochemically. The Au/nAu electrode was transferred to a solution of KCl (0.5 mol dm^{-3}) containing $K_4[Fe(CN)_6]$ (0.5 mmol dm^{-3}) and cyclic voltammograms at different potential sweep rates were measured. Using the Randles-Sevcik equation (Bard et al., 2001) and the value of 7.60×10^{-6} cm s^{-1} for the diffusion coefficient of $[Fe(CN)_6]^{4-}$ (Wang et al., 2007), we obtained the real surface area of the Au/nAu electrode.

OCP measurements of the Au/nAu electrode during the self assembling process of p-ssDNA were performed using a digital voltmeter of MS8340B Digital Multimeter, Mastech (China) connected to a PC and an Au/nAu screen-printed electrode refrigerated during the measurements.

Electrochemical detection of the DNA hybridization was performed in an electrochemical cell containing 10 mL Tris by recording cyclic voltammograms (CVs) and differential pulse voltammograms (DPVs) for the reduction peak of intercalated MB. The parameters of DPV recording were pulse width of 25 mV, a pulse time of 50 ms, and a scan rate of 10 mV s^{-1}. The concentration of t-ssDNA was quantified as the MB reduction peak current.

Bacteria culture, human samples, and genomic DNA extraction

Brucella agar culture medium with the addition of 5% horse blood was used for the cultivation of *Brucella* organisms by incubation at 35±2 °C for 48-72 h.

7 human serum samples with Brucellosis (Wright and 2ME tests were positive in all samples) were provided from veterinary administration of Fars province. It should be noted that a healthy blood sample was evaluated with suspicious samples. From 7 human serum samples with Brucellosis, 6 were measured after amplification by PCR and one sample was measured without amplification. Before performing the required investigations, the genomes were placed for 5 min at 90 ° C.
